# Impact of heat waves on human morbidity and hospital admissions in a city of the western mediterranean area

**DOI:** 10.1007/s00420-024-02082-y

**Published:** 2024-07-02

**Authors:** Adrián Bujosa Mateu, Luis Alegre Latorre, María Villalonga Comas, Jaume Salom, Mercedes García Gasalla, Lluis Planas Bibiloni, Jaime Orfila Timoner, Javier Murillas Angoiti

**Affiliations:** 1 Department of Medicine, Universidad de Las Islas Baleares, Palma, Spain; 2 IREC Catalonia Institute for Energy Research, Barcelona, Spain; 3 Internal Medicine Department, Hospital Universitario Son Espases, Palma, Spain; 4 Instituto de Investigación de Las Islas Baleares Idisba, Palma, Spain

**Keywords:** Climate change, Heat wave, Hospital admissions, Critical care unit admissions, Heat stroke, Morbidity

## Abstract

**Purpose:**

The effect of heat waves on mortality is well known, but current evidence on morbidity is limited. Establishing the consequences of these events in terms of morbidity is important to ensure communities and health systems can adapt to them.

**Methods:**

We thus collected data on total daily emergency hospital admissions, admissions to critical care units, emergency department admissions, and emergency admissions for specific diagnoses to Hospital Universitario de Son Espases from 1 January 2005 to 31 December 2021. A heat wave was defined as a period of ≥ 2 days with a maximum temperature ≥ 35 °C, including a 7 day lag effect (inclusive). We used a quasi-Poisson generalized linear model to estimate relative risks (RRs; 95%CI) for heat wave-related hospital admissions.

**Results:**

Results showed statistically significant increases in total emergency admissions (RR 1.06; 95%CI 1 – 1.12), emergency department admissions (RR 1.12; 95%CI 1.07 – 1.18), and admissions for ischemic stroke (RR 1.26; 95%CI 1.02 – 1.54), acute kidney injury (RR 1.67; 95%CI 1.16 – 2.35), and heat stroke (RR 18.73, 95%CI 6.48 – 45.83) during heat waves.

**Conclusion:**

Heat waves increase hospitalization risk, primarily for thromboembolic and renal diseases and heat strokes.

## Introduction

It is widely accepted by the general scientific community that climate change has caused an increase in global temperature. The average temperature has increased by more than 1.2 °C since the pre-industrial era, and climate projections predict an increase above 2 °C in the coming decades if effective mitigation measures are not applied (Watts et al. 2021). Climate change is also increasing both the frequency and the intensity of extreme weather events such as heat waves (Ebi et al. 2021). Identifying the impact of heat waves on people’s health is a priority in the adaptation process of communities and health systems, particularly given future climate scenarios and the increase in vulnerable populations resulting from demographic ageing in industrialized countries.

Many studies have shown the effect of these extreme heat events on population mortality. The MoMo (daily all-cause mortality monitoring system, by its acronym in Spanish) report published annually by the Instituto de Salud Carlos III in Spain estimated that in 2021 a 1.22% increase in mortality attributable to high temperatures occurred. Since this report started being published, 2021 was the year with the smallest impact on mortality whereas 2003 was the year in which a 6.98% excess mortality rate associated with a higher temperature was found (Leon et al. 2021). However, evidence of an association between morbidity and heat waves is still limited and studies often provide inconsistent results (Li et al. 2015). For example, a study conducted in the United States analyzing the increase in hospital admissions during extreme heat events showed increased admissions for renal diseases while admissions for respiratory diseases did not significantly vary and admissions for cardiovascular diseases decreased (Gronlund et al. 2014). Similar findings were obtained in a study where hospital admissions for cardiovascular and respiratory diseases were investigated during heat waves in 12 European cities (Michelozzi et al. 2009). It is also worth mentioning that most studies seem to show a greater impact on mortality than on morbidity. Certain authors attribute this difference to a “harvest effect” whereby the most vulnerable patients die before being able to access the health system (Zhao et al. 2019; Kovats et al. 2004), although there may be confounding social and health factors given the heterogeneity of the published results. Cardiovascular, pulmonary, and renal diseases are among the diseases affected by acute increases in temperature for which there is the highest degree of scientific evidence.(Ebi et al. 2021; Székely et al. 2015; Kovats et al. 2004; Cicci et al. 2022; Witt et al. 2015; Gostimirovic et al. 2020). However, temperature increases have been shown to have a transversal effect on health, affecting all body systems, and therefore to have an influence on the occurrence of multiple illnesses of neurospsychiatric (Thompson et al. 2018; Liu et al. 2018), infectious (Gostimirovic et al. 2020; Gupta et al. 2016; Ghazani et al. 2018), and endocrine-metabolic origin (Hopp et al. 2018) among others. Although these effects can be seen at any age, the elderly population constitutes the most vulnerable group. A reduced thermoregulatory ability, increased comorbidity, polypharmacy, social isolation, and dependence are some of the factors primarily involved in the increased risk found in this population subgroup (Bouchama et al. 2007). Nevertheless, the consequences of exposures to extreme heat events are also affected by other factors such as the place of residence, socioeconomic level, job type, or substance use. (Ebi et al. 2021; Ozturk 2023).

This study was designed to provide a better understanding of the effects of extreme heat waves on human health. Its aim was to determine the effect of heat waves on morbidity in the population of a healthcare area (*Sector Sanitario de Ponent*) located in the island of Mallorca, in Spain.

## Material and methods

The study was conducted in the city of Palma de Mallorca in the Balearic Islands, in the Mediterranean Sea, where the climate is typically Mediterranean with temperate temperatures in winter and characteristically hot and humid summers, although precipitations are scarce and irregular. The healthcare area under study was the Ponent area, with a population of 346,834 inhabitants. Because the local economy is primarily based on tourism, important seasonal changes in population numbers occur and the total population may increase by up to 74% during the summer months. In accordance with the Spanish National Health System regulations, the current healthcare model is based on a universal public healthcare system and the study was carried out in that context.

Data from daily individual emergency admissions to Hospital Universitario de Son Espases (previously named Hospital Universitario Son Dureta until 2011) from 1 January 2005 to 31 December 2021 as well as data from daily individual admissions to critical care units (CCUs) and emergency department admissions from 1 January 2016 to 31 December 2021 were collected from the hospital discharge minimum dataset (*Conjunto Mínimo Básico de Datos de las Altas Hospitalarias*). The total number of daily emergency admissions (total admissions to hospital), admissions to CCUs, and emergency department admissions (admissions to emergency department) were also collected from this dataset. Information on patient sex, age, and postal code (PC) for each admission was obtained to derive the total number of daily emergency admissions by sex (man or woman), age (stratified into four groups: < 15 years, 15–65 years, 66–80 years, and > 80 years), and PC. PC is a unit of administrative division of the population, and it was used as a reference to analyze emergency admissions by population type (urban vs. rural) and average gross income (AGI). Admission PCs were classified as “urban” or “rural” based on the definition used in the 2020 report on Spanish Urban Areas of the Ministry of Transport, Mobility, and Urban Agenda (Ministerio de Transportes 2021). Data on AGI by PC were obtained from the Spanish Tax Agency’s official web site, which provides personal income tax statistics by PC for the larger municipalities (AgenciaTributaria). Palma de Mallorca’s PCs were divided into three subgroups (low income, high income, and middle income), with 25% of PCs having a low AGI, 25% a high AGI, and the remaining 50% a middle AGI.

Daily individual admissions stratified by primary and secondary diagnosis of diseases for which the impact of heat waves had previously been described in other studies were also collected based on the ICD-9 coding system (until 2015) and the ICD-10 (from 2016 to 2021). Admissions for specific diagnoses included the following six nosologic groups: heat stroke, cardiovascular diseases (acute myocardial infarct, angina, heart failure (HF) exacerbation, arrhythmias, pulmonary thromboembolism (PTE), deep venous thrombosis (DVT), syncope, hypotension, hypertension crisis, hypertension), pulmonary diseases (chronic obstructive pulmonary diseases [COPD] exacerbation, asthma exacerbation, acute respiratory distress syndrome [ARDS]), acute respiratory failure), neurological diseases (ischemic stroke, hemorrhagic stroke, seizure, Parkinson disease, multiple sclerosis), reno-urological diseases (acute kidney injury [AKI], chronic kidney disease (CKD) exacerbation, upper urinary tract stones), and infectious diseases (gastrointestinal infections, pneumonia). Data were processed to obtain the total daily number of admissions by primary diagnosis. However, we decided to include in our analysis diseases that can be underrepresented at admission, such as “exacerbation of COPD” for which the primary diagnosis may be recorded as “pneumonia” while the code indicating “exacerbation of COPD” may be recorded as secondary diagnosis, irrespective of whether they were recorded as primary or secondary diagnosis. These were HF exacerbation, PTE, hypertension, hypotension, hypertension crisis, COPD exacerbation, asthma exacerbation, ARDS, CKD exacerbation, Parkinson disease, and multiple sclerosis.

### Meteorological data

Maximum, minimum, and mean daily temperatures in Palma de Mallorca (Palma-Puerto weather station) were obtained from the OpenData system of the Spanish Meteorological Agency (*Agencia Estatal de Meteorología*—AEMET) from 1 January 2005 to 31 December 2021. Daily data on suspended particles (PM10, inhalable particulate matter with diameters between 10 µ and 2,5 µm) and tropospheric ozone (O3) concentrations in Palma de Mallorca (Palma-Foners station) from 1 January 2005 to 31 January 2021 were obtained from the Climate Change and Atmosphere Service (*Servicio de Cambio Climático y Atmósfera*) of the Regional Ministry of Energy Transition, Productive Sectors, and Democratic Memory (*Consejería de Transición Energética, Sectores Productivos y Memoria Democrática*). Atmospheric pollution indicators (PM10 and O3) have been considered as confounding factors in previous studies and were therefore adjusted in the statistical analysis.

### Definitions

Currently there is no agreed definition of heat wave (HW) in the scientific community, but there are a series of recommendations to develop a definition (Cicci et al. 2022; Xu et al. 2016; Honda et al. 2014). On the one hand, no significant differences have been found between the use of mean temperature (Tmean) or maximum temperature (Tmax) as a reference unit, although it is advisable to apply a temperature threshold for which an impact on the health of the study population has been evidenced in previous analyses. On the other hand, there is a certain degree of consensus in similar studies to apply a minimum period of 2 consecutive days of temperatures above the threshold to define a period as a HW.

In line with these recommendations, we defined a HW as a level of heat starting at 1 °C below the temperature threshold for the Balearic Islands at which there is a statistically significant increase in mortality. This threshold was determined in the study by Díaz Jiménez, et al. on trigger threshold temperatures for mortality attributable to heat in Spain during the 2000–2009 period (Díaz Jiménez J 2015). This study is currently considered as the consensus study for further research on the heat-related health impact in Spain. We decided to lower the temperature threshold by 1 °C to increase the number of HW events and thus obtain a more homogeneous distribution of these events during the study period. Therefore, in our study, a HW was defined as a period ≥ 2 consecutive days with Tmax ≥ 35 °C.

Patients were considered to have been admitted for exposure to a HW when they were admitted to Hospital Universitario de Son Espases during one of the periods defined as HWs (based on the definition described above) or until 7 days (inclusive) following a HW episode. This was to ensure a time window from the onset of pathophysiological effects secondary to high temperatures to patient admission, with a definitive primary diagnosis. This phenomenon is called “lag effect” (Li et al. 2015; Honda et al. 2014).

### Statistical analysis

The data collected from the daily admissions to Hospital Universitario de Son Espases (previously called Hospital Son Dureta) during the period 2005–2021, both inclusive, were analyzed. A quasi-Poisson distribution was used with a generalized linear model (GLM) to estimate relative risks (together with their 95%CIs) associated with HWs compared to periods of no-HWs for each study variable separately. The analysis was performed using the programming language Rv4.2.2 and was as follows for each study variable:

Quasi-Poisson (X)—> glm(αf ~ HWf + fechaf + o3f + pm10f, data = Y, family = quasipoisson(link = “log”)).

where X is the study variable, αf is the number of admissions with the study variable on day f, and HWf is an independent binary variable identifying if day f is within a HW or a no-HW period (1 and 0, respectively). The factor fechaf is an independent ordinal variable identifying the day, month, and year of the day f to control for changes in trends in admissions over time. Additionally, o3f and pm10f are independent quantitative variables representing the level of pollution on day f based on the concentration of O3 and the concentration of suspended particles in the air in micrograms per cubic meter (μg/m3). Thus, the analysis was adjusted to these parameters that were assumed to act as confounding factors. Lastly, data = Y provides information on the source from which the data had to be extracted, and the function family = quasipoisson identifies the distribution to which the analysis was adjusted. All statistical tests were two-sided and values of p < 0.05 were considered statistically significant.

Study variables were the items already described above: total emergency admissions, total CCU admissions, emergency department admissions, sociodemographic variables, and admissions with specific diagnoses. As mentioned, the sociodemographic variables recorded (sex, age, and PC) were divided into 2, 4, and 5 study variables respectively (man and woman for sex; < 15 years, 15–65 years, 66–80 years, and > 80 years for age; low income, middle income, high income, urban, and rural for PC).

## Results

Based on the definition described earlier, a total of 6 HWs were identified during the study period (1 January 2005 – 31 December 2021), all during the months of July and August. The total number of days analyzed was 6809, among which 58 were considered exposure days to a HW, including the 7 days following a HW to compensate for the lag effect. Table 1 (see Annex) shows the average values of the mean, maximum, and minimum temperatures during the study period, including the periods of no-HWs and HWs. The difference between average temperatures over the whole study period and during the HW periods is of note.

**Table 1:** Average values of mean, maximum, and minimum temperatures during HW and no-HW periods

	T mean (°C)	T max (°C)	T min (°C)
No-HW	18.6	22.3	14.9
HW	28.6	32.5	24.7
Total	18.7	22.4	15

*HW* heat wave, *T* temperature

During this period, there was a total of 287,609 emergency admissions. Among these, 284,718 were admissions during no-HWs and 2891 during HWs, approximately 1% of the admissions. The characteristics of admitted patients is summarized in Table 2.

**Table 2:** Emergency admissions during the study period

	Emergency admissions
No-HW	284,718
HW	2891
Total	287,609

	%
Male	48.8
Female	51.2
< 15 y/o	12.1
15–65 y/o	50.3
65–80 y/o	22.3
> 80 y/o	15.3

**Fig. 1:**
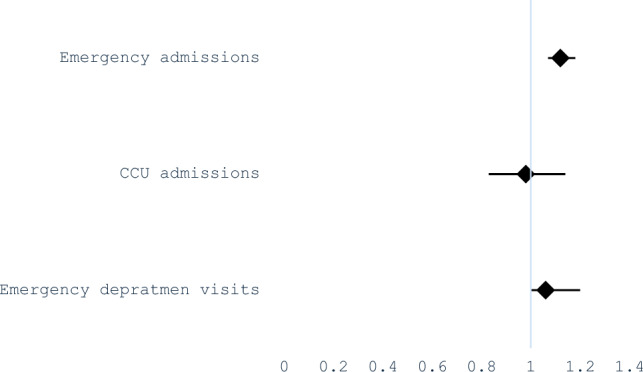
Relative risks of admission and emergency visits during HW

The relative risk (RR) for total emergency admissions associated with HWs compared to no-HWs was 1.06 (95%CI 1 – 1.12). In addition, a significant increase in emergency department admissions was found during HW compared to no-HWs (RR 1.12; 95%CI 1.07 – 1.18). However, we were unable to establish an association between CCU admissions and HW periods (RR 0.98; 95%CI 0.83 – 1.14). These results are depicted in Fig. 1 and values are summarized in Table 3.

**Table 3:** Relative risks (95% CI) for total emergency admissions, CCU admissions, and emergency department visits

	RR	95% CI
Emergency admissions	1.06	11.12
CCU admissions	0.98	0.831.14
Emergency department visits	1.12	1.071.18

*CCU* critical care unit, *RR* relative risk

**Fig. 2:**
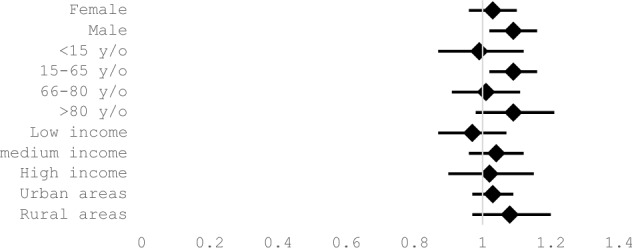
Sociodemographic variables relatives risk of admissions associated with HW

The results for the sociodemographic variables (Table 4) studied showed the existence of a statistically significant risk associated to HWs in men, with an RR of 1.09 (95%CI 1.02 – 1.16). For age, the only segment for which results showed a significant increase in the risk of admission associated to HWs was found in the population subgroup aged between 15 and 65 years (RR 1.09; 95%CI 1.02 – 1.16), which is representative of the active population at the time of admission. No significant differences were found between urban and rural areas or between the level of income based on the PC. (Fig. 2).

**Table 4:** Relative risks of HW-related admissions for sociodemographic variables

Variables	RR	95% CI
Female	1.03	0.961.1
Male	1.09	1.021.16
< 15 y/o	0.99	0.871.12
15–65 y/o	1.09	1.021.16
66–80 y/o	1.01	0.911.11
> 80 y/o	1.09	0.981.21
Low income	0.97	0.871.07
Medium income	1.04	0.961.12
High income	1.02	0.91.15
Urban area	1.03	0.971.09
Rural area	1.08	0.97–1.2

*HW* heat wave, *RR* relative risk, *y/o* years old

**Fig. 3:**
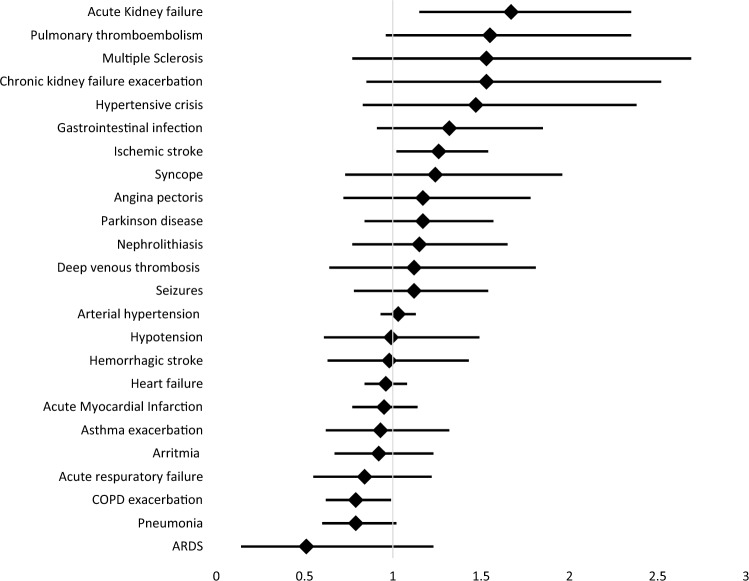
Specific diagnoses admission risks

Lastly, admission risks for specific diagnoses associated to HWs are provided in Table 5. As seen in Fig. 3, there was a statistically significant increased risk of admission associated to HWs for ischemic stroke (RR 1.26; 95%CI 1.02 – 1.54), AKI (RR 1.67; 95%CI 1.16 – 2.35), and heat stroke (RR 18.73, 95%CI 6.48 – 45.83). In addition, it should be noted that the 95%CI for the diagnosis of PTE is at the limit of the statistical significance (RR 1.55; 95%CI 0.96 – 2.35), in line with findings obtained for other events of thromboembolic origin such as ischemic stroke. Furthermore, the trends in admission risks associated to HWs for respiratory diseases appear to have been negative (asthma exacerbation [RR 0.93; 95%CI 0.62 – 1.32], acute respiratory failure [RR 0.84; 95%CI 0.55 – 1.22], pneumonia [RR 0.79; 95%CI 0.6 – 1.02], COPD exacerbation [RR 0.79; 95%CI 0.62 – 0.99], and ARDS [RR 0.51; 95%CI 0.14 – 1.23]) and, in fact, it appears that HWs may act as a protection factor for the “exacerbation of COPD” diagnosis.

**Table 5:** Admission risks for specific diagnoses

Diagnosis	RR	95% CI
ARDS	0.51	0.14–1.23
Pneumonia	0.79	0.6–1.02
COPD exacerbation	0.79	0.62–0.99
Acute respiratory failure	0.84	0.55–1.22
Arrythmia	0.92	0.67–1.23
Asthma exacerbation	0.93	0.62–1.32
Acute myocardial Infarction	0.95	0.77–1.14
Heart failure	0.96	0.84–1.08
Hemorrhagic stroke	0.98	0.63–1.43
Hypotension	0.99	0.61–1.49
Hypertension	1.03	0.93–1.13
Deep venous thrombosis	1.12	0.64–1.81
Seizures	1.12	0.78–1.54
Nephrolithiasis	1.15	0.77–1.65
Angina pectoris	1.17	0.72–1.78
Parkinson disease	1.17	0.84–1.57
Syncope	1.24	0.73–1.96
Ischemic stroke	1.26	1.02–1.54
Gastrointestinal infection	1.32	0.91–1.85
Hypertensive crisis	1.47	0.83–2.38
Multiple sclerosis	1.53	0.77–2.69
Chronic kidney failure exacerbation	1.53	0.85–2.52
Pulmonary thomboembolism	1.55	0.96–2.35
Acute kidney failure	1.67	1.15–2.35
Heat stroke	18.73	6.48–45.83

*ARDS* acute respiratory distress syndrome, *COPD* chronic obstructive pulmonary disease, *RR* relative risk

## Discussion

Our study has identified a statistically significant risk for total emergency admissions (RR 1.06; 95%CI 1 – 1.12) and admissions to the emergency department (RR 1.12; 95%CI 1.07 – 1.18) associated to HWs. However, an association between HWs and CCU admissions could not be found (RR 0.98; 95%CI 0.83 – 1.14). Most recent studies coincide in showing the existence of increased hospitalizations during HW periods. A systematic review reported a 2–11% increase in admissions during HWs and also identified an increase in emergency department admissions in most studies where this variable was investigated (Li et al. 2015). Nevertheless, in a meta-analysis evaluating mortality and morbidity jointly in extreme temperatures, it was seen that while an increase in all-cause mortality occurred, hospital admissions increased only for certain diagnoses (Bunker et al. 2016). Furthermore, evidence on increased CCU admissions during HWs is still very limited.

Our analysis of the risk of admission associated to HWs by sex shows the existence of a statistically significant risk of admission for men during HWs compared to no-HWs (RR 1.09; 95%CI 1.02 – 1.16). Differences between sexes regarding the impact of HWs on health have been identified in multiple studies, although their findings are heterogeneous and both other studies and this study suggest that the differences found between sexes are more due to ethnic or sociodemographic characteristics of each specific community than to intrinsic differences in the physiology of both sexes (Li et al. 2015; Zhao et al. 2019; Oudin Åström et al. 2015).

Regarding age, we found a statistically significant risk of admission for the active population age range (RR 1.09; 95%CI 1.02 – 1.16). Although there seems to be a positive trend in RR for older subgroups (66–80 and > 80 years), we cannot confirm the existence of a higher admission risk during HWs in our study. Our study findings may be interpreted in two ways. On the one hand, a greater environmental exposure to heat in the active population resulting from typical work activity in the Balearic Islands could explain the increased risk of admission in this population subgroup during HWs. In contrast, the population considered as more vulnerable may tend to protect itself from the heat during these events, thus avoiding their primary impacts. On the other hand, certain studies suggest there may be a “harvest effect” where an increased in out-of-hospital mortality may be hiding an increase in hospital admissions (Zhao et al. 2019; Hopp, Dominici, and; Bobb 2018, Bobb et al. 2014).

The remaining sociodemographic variables assessed in our study were population type (urban or rural) and AGI. Although urban populations are assumed to be more affected during HWs compared to inhabitants in rural areas because of the so-called “urban heat island” effect, we did not find significant differences in our study and neither did we find significant differences by income level given that postal codes are probably not an adequate stratification measure to differentiate income levels.

The most common diagnosis leading to hospital admissions was heat stroke, which represented the diagnosis with the highest RR associated to HWs in our study (RR 18.73, 95%CI 6.48 – 45.83). Two types of heat strokes are recognized based on the predisposing event: a classic or passive heat stroke occurs in persons with a compromised thermoregulatory capacity (extreme age, use of anticholinergic agents, ) while an active heat stroke occurs when the performance of physical activities involves exposure to high temperatures (Székely et al. 2015). Irrespective of the type of heat stroke, the increase in environmental temperature and, particularly, the increase occurring during HWs constitutes the primary precipitating factor for the development of this entity (Hopp, Dominici, and Bobb 2018; Bobb et al. 2014; Kenney et al. 2014). It should be noted, however, that although heat strokes represent the primary direct cause of death due to extreme weather events, it only accounts for a minority of deaths attributable to HWs (Kenney et al. 2014).

Secondly, AKI showed a significant increased risk of admission associated to HWs compared to no-HWs (RR 1.67; 95%CI 1.16 – 2.35). Pathophysiologically, dehydration, electrolyte imbalance, and vascular redistribution secondary to a temperature increase can induce renal stress (Li et al. 2015). Additionally, plasma hyperosmolarity and an increased vasopressin secretion can damage the glomerular complex, ultimately leading to an AKI (Gostimirovic et al. 2020). This finding is consistent with prior evidence showing how extreme heat events increase hospitalization risk for renal diseases, in particular for AKI (Zhao et al. 2019; Kovats et al. 2004). This impact has been observed not only during extreme heat periods but also during events of moderate temperatures when admissions for illnesses of renal origin, mainly in vulnerable population, can also increase (Gronlund et al. 2014).

Thirdly, there was a statistically significant risk of hospitalization during HWs for ischemic strokes (RR 1.26; 95%CI 1.02 – 1.54). Studies on morbidity associated to HWs provide inconsistent results for cerebrovascular diseases. A systematic review conducted by R. Cicci et al. revealed that the majority of studies where cerebrovascular events were jointly analyzed (ischemic and hemorrhagic strokes), non-significant risks for admissions during HWs were obtained. In contrast, studies analyzing both stroke types separately have generally found statistically significant increases in hospitalization risk for ischemic strokes (Cicci et al. 2022). A meta-analysis conducted on the elderly population has also obtained results pointing to a “harvest effect” as mentioned earlier. This article indicated the existence of a lag time between the increase in deaths and hospital admissions due to cerebrovascular diseases during HWs (Bunker et al. 2016). The increase in ischemic stroke is biologically plausible since the increase in body temperature can induce changes in blood composition (increase in viscosity, erythrocyte concentration and cholesterol levels) as well as in the maintenance of blood pressure (vascular redistribution towards the peripheral circulation) that could decrease cerebral blood flow. These effects increase the concentration of platelets above what would be expected due to hemoconcentration derived from fluid loss and modify their activity, increasing their thrombogenic capacity.

We were not able to establish a statistically significant increased risk of admission associated to HWs for the cardiovascular disease group (acute myocardial infarct, angina, HF exacerbation, arrythmias, PTE, DVT, syncope, hypotension, hypertension crisis, and hypertension). However, several aspects of our findings are of interest. First, PTE was the diagnosis of cardiovascular origin with the highest RR and a lower limit of the 95%CI closer to statistical significance (RR 1.55; 95%CI 0.96 – 2.35). Current evidence associating PTE with extreme heat events is small, but several studies point towards an increased incidence during extreme HWs, and this is biologically plausible given the pathophysiological changes associated with extreme heat (LW 2022; Keatinge et al. 1986). Furthermore, another aspect worth mentioning regarding diseases of cardiovascular origin in our study is that acute myocardial infarcts (RR 0.95; 95%CI 0.77 – 1.14), HF exacerbations (RR 0.96; 95% CI 0.84 – 1.08), and arrythmias (RR 0.92; 95% CI 0.67 – 1–23) had RRs for admission during HWs below the neutral value. Although these risks were not statistically significant, this negative association between cardiac diseases and extreme heat events has already been observed in other studies on morbidity (Zhao et al. 2019; Bobb et al. 2014). These findings could partly be explained by the “harvest effect” mentioned, as the increase in out-of-hospital mortality could reduce expected admissions during extreme heat events (Kovats et al. 2004; Bunker et al. 2016).

Lastly, the diagnoses of respiratory origin included in our study were asthma exacerbation, COPD exacerbation, ARDS, and acute respiratory failure. When comparing this nosologic group with the rest of diagnoses assessed, a negative trend in the association between admission risk for these entities and HW periods was revealed. COPD exacerbation even showed a statistically significant negative RR of admission associated with HWs (RR 0.79; 95%CI 0.62 – 0.99). The evidence obtained in studies on morbidity and mortality studies due to respiratory diseases during such weather events is conflicting (Michelozzi et al 2009 and Kovats eta al 2004). A potential explanation might be the need to include atmospheric pollution as a covariable that could explain the increased respiratory diseases during HWs (Bunker et al. 2016). Cerro et al. (2015) evidenced and interpreted interesting downward trends for ambient concentrations of pollutants over the Balearic Islands in the period 2000–2013, more pronounced at urban sites (1.0 µg/m3 annual PM10 decrease) than at the regional background (0.6 µg/m3 annual PM10 decrease). Moreover, prominent O3 maximum is recorded in spring over the Balearic Islands, instead of a summer maximum as usually observed at continental sites in the Western Mediterranean basin. (Cerro et al 2015).

## Conclusions

Extreme HWs, which are becoming more common as part of extreme meteorological events linked to global warming, were associated in our study with increased hospital admissions and increased visits to the emergency department during HW compared to no-HWs, but not to an increase in intensive care unit admissions.

Diagnoses at discharge increasing during HWs were heat stroke, AKI, and ischemic stroke.

The impact of HWs on morbidity was greater in men aged 15 to 65 years.
